# Feasibility of Video-Assisted Thoracoscopic Surgery via Subxiphoid Approach in Anterior Mediastinal Surgery: A Meta-Analysis

**DOI:** 10.3389/fsurg.2022.900414

**Published:** 2022-05-06

**Authors:** Yuxiang Luo, Feng He, Qingchen Wu, Haoming Shi, Dan Chen, Hongtao Tie

**Affiliations:** ^1^ Department of Cardiothoracic Surgery, The First Affiliated Hospital of Chongqing Medical University, Chongqing, China; ^2^ Department of Cardiothoracic Surgery, Fifth People’s Hospital of Chongqing, Chongqing, China

**Keywords:** subxiphoid video-assisted thoracoscopic surgery, subxiphoid approach, thymoma, meta-analysis, anterior mediastinal surgery

## Abstract

**Background:**

Accumulating researches show potential advantages of video-assisted thoracoscopic surgery (VATS) via the subxiphoid approach, and this meta-analysis aims to investigate the efficacy and safety of the subxiphoid approach for anterior mediastinal surgery.

**Methods:**

Relevant studies were retrieved by searching Embase and PubMed databases (from the inception to October 1, 2021). Primary outcomes included postoperative pain, intraoperative blood loss, operation time, chest tube duration, and hospital length of stay. All meta-analyses were performed by using random-effects models.

**Results:**

Overall, 14 studies with 1,279 patients were included, with 504 patients undergoing anterior mediastinal surgery via subxiphoid approach and 775 via other approaches. The pooled results indicated that the subxiphoid approach was associated with reduced postoperative pain indicated by visual analog scale [weight mean difference (WMD): 24 h: −2.27, 95% CI, −2.88 to −1.65, *p* < 0.001; 48–72 h: −1.87, 95% CI, −2.53 to −1.20, *p* < 0.001; 7 days: −0.98, 95% CI, −1.35 to −0.61, *p* < 0.001], shortened duration of chest tube drainage (WMD: −0.56 days, 95% CI, −0.82 to −0.29, *p* < 0.001), shortened hospital length of stay (WMD: −1.46 days, 95% CI, −2.28 to −0.64, *p* < 0.001), and reduced intraoperative blood loss (WMD: −26.44 mL, 95% CI, −40.21 to −12.66, *p* < 0.001) by comparison with other approaches in anterior mediastinal surgery. Besides, it has no impact on operation time and the incidence of complications of transition to thoracotomy, postoperative pleural effusion, phrenic nerve palsy, and lung infection.

**Conclusions:**

Our study suggests that the subxiphoid approach is a feasible alternative approach and even can be a better option for anterior mediastinal surgery. Further, large-scale multicenter randomized controlled trials are needed to validate this finding.

## Introduction

Video-assisted thoracoscopic surgery (VATS) brings relevant achievements, including reducing surgical trauma with lesser acute and chronic pain, shortening hospital stay, decreasing morbidity, having a better cosmetic effect, and fastening the recovery time for thoracic surgery (1). However, different approaches exist for VATS, including unilateral (right- or left-sided), bilateral, subxiphoid, transcervical, or combined approaches (2). Among them, VATS via the subxiphoid approach shows some advantages: avoiding intercostal nerve damage, decreasing postoperative pain, decreasing blood loss, bringing better cosmetic effects, and shortening the surgical duration and hospital stay (3). Moreover, simultaneous access to both pleural cavities is provided in the subxiphoid approach, which greatly improves the view by split-lung ventilation and facilitates the complete removal of the anterior mediastinal lesions (4). However, accumulating research reported inconsistent effects of the subxiphoid approach in patients undergoing anterior mediastinal surgery. Therefore, we performed the current meta-analysis to explore the role of the subxiphoid approach in anterior mediastinal surgery.

## Methods

This meta-analysis was reported following the Preferred Reporting Items for Systematic Reviews and Meta-Analyses (PRISMA) Statement (5). We registered this meta-analysis at the International Platform of Registered Systematic Review and Meta-analysis Protocols (https://inplasy.com/), with the registered ID: INPLASY202230031.

### Search Strategy and Selection Criteria

We conducted relevant studies by searching Embase and PubMed databases (from the inception to October 1, 2021). Two investigators (HT, FH) performed the literature search independently. The reference list of retrieved studies and related reviews were also manually screened, and the process was performed repeatedly until no further eligible studies were included. We regarded studies as eligible for inclusion according to the following criteria: (1) Patients with anterior mediastinal lesion receiving surgery therapy; (2) Subxiphoid approach as intervention group; (3) Control group including any other approaches; and (4) Cohort study or randomized controlled trials. The preliminary selection was performed by screening titles and abstracts, and then full-text articles were read to select the eligible ones. Study selection was performed by two investigators independently (HT, FH).

### Data Extraction and Quality Assessment

We extracted the following information from each included study: first author, year of publication, study area, study design, recruitment period, patients’ type, number of patients in the intervention group, number of patients in the control group, and description of the subxiphoid approach and the control. The quality of each study was evaluated using the modified Newcastle–Ottawa quality scale, with a maximum of 9 points (6). The higher scores mean a lower risk of bias. Two independent investigators (HT, FH) accomplished the data extraction and quality assessment.

### Outcome Measure

We treated postoperative pain, intraoperative blood loss, operation time, chest tube duration, and hospital length of stay (HLOS) as primary outcomes. Secondary outcomes were the incidence of complications of transition to thoracotomy, postoperative pleural effusion, phrenic nerve palsy, and lung infection.

### Statistical Analysis

Differences were expressed as weighted mean differences (WMDs) with 95% confidence intervals (CIs) for the primary outcomes and relative risks (RRs) with 95% confidence intervals (CIs) for the secondary outcomes. Heterogeneity among the included studies was detected by using the *I*^2^ statistic and evaluated as low (25%–50%), moderate (50%–75%), and high heterogeneity (greater than 75%) (7). All meta-analyses were performed by using random-effects models. Sensitivity and subgroup analyses according to area and control group were performed. Publication bias was only evaluated if more than ten studies were included for the primary outcomes according to our previous publication (8). A two-tailed *p* < 0.05 was considered as a significant level. Because of the zero-events studies, the statistical analyses were performed using RevMan 5.1 for the primary outcomes and R software for the secondary outcomes.

## Results

### Literature Search and Study Selection

**Figure 1:**
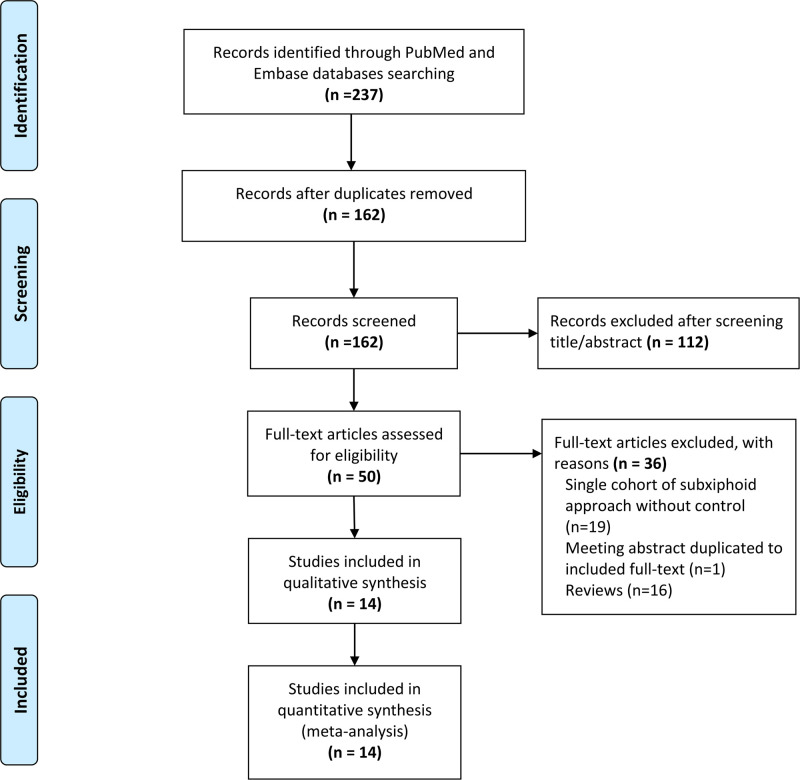
Flow chart of the selection process.

The flow chart of the detailed screening process is shown in Figure 1. Overall, 237 studies were searched from PubMed and Embase databases. After abstract/title screening and full-text reading, 14 eligible studies were finally included in our meta-analysis according to the selection criteria (9–22).

### Characteristics of Eligible Studies

The basic characteristics of included studies are summarized in Table 1, and the detailed description of the subxiphoid and control approaches is listed in Supplementary Table S1. Besides, the outcome data are presented in Supplementary Tables S2–S4. Fourteen studies involving 1,279 patients were included, with ten performed in China and four in Japan. Among them, 504 patients received anterior mediastinal surgery via the subxiphoid approach as the intervention group, and 775 patients received anterior mediastinal surgery via other approaches as the control group. Among the included studies, six enrolled patients with myasthenia gravis, four enrolled patients with Masaoka stage I–II thymoma without myasthenia gravis, three studies enrolled patients with anterior mediastinal tumor or myasthenia gravis, and one study enrolled patients with Masaoka stage I–II thymoma, hyperplasia of the thymus gland with myasthenia gravis, or thymic cysts. Anterior mediastinal surgery via the subxiphoid approach was achieved through an incision below the xiphoid and two ports under the bilateral costal arches in most studies, and the uniportal subxiphoid approach or modified subxiphoid uniportal approach was used in five studies (12, 13, 18, 20, 21). In the control group, unilateral thoracoscopic approach and median sternotomy were used. Of all 14 studies, one is prospective cohort study, and the others are retrospective cohort studies. NOS scores range from 6 to 8, suggesting a moderate-to-good quality of each study.

**Table 1:** Baseline characteristics of included studies.

Study ID	Area	Study design	Recruitment period	No.	Patients	Comparison		Study quality (NOS Score)
				Subxiphoid/control		Subxiphoid approach	Control	
Cao 2022	China	Retrospective cohort	January 2013 to December 2019	65/72	Patients with myasthenia gravis	Subxiphoid and subcostal arch approach	Unilateral thoracoscopic approach	6
Hsu 2004	China	Prospective cohort	March 2001 to February 2003	15/12	Patients with myasthenia gravis	Subxiphoid bilateral thoracoscopic approach	Right-side thoracoscopic approach	7
Jiang 2021	China	Retrospective cohort	January 2015 to May 2019	39/198	Masaoka stage I and II thymoma without myasthenia gravis	Subxiphoid and subcostal arch approach	Unilateral thoracoscopic approach	6
Liu 2021	China	Retrospective cohort	August 2015 to September 2019	76/76	Masaoka stage I and II thymoma without myasthenia gravis	Subxiphoid uniportal approach	Intercostal uniportal VATS	8
Lu 2018	China	Retrospective cohort	December 2012 to December 2014	41/36	Myasthenia gravis with or without Masaoka stage I and II thymoma	Subxiphoid and subcostal arch approach	Right-side thoracoscopic approach	6
Qiu 2020	China	Retrospective cohort	January 2013 to October 2017	68/63	Patients with myasthenia gravis	Subxiphoid and subcostal arch approach	Right-side unilateral thoracoscopic approach	6
Shiomi 2018	Japan	Retrospective cohort	January 2010 to December 2016	13/20	Myasthenia gravis with or without Masaoka stage I and II thymoma	Modified subxiphoid uniportal approach	Median sternotomy	6
Suda 2016	Japan	Retrospective cohort	January 2005 to December 2014	46/35	Anterior mediastinal tumor or myasthenia gravis	Subxiphoid uniportal approach	Thoracoscopic approach	6
Tang 2015	China	Retrospective cohort	April 2014 to June 2015	20/25	Patients with myasthenia gravis	Subxiphoid and subcostal arch approach	Right-side thoracoscopic approach	6
Wang 2017	China	Retrospective cohort	January 2015 to December 2016	36/47	Early-stage thymomas without myasthenia gravis	Modified subxiphoid uniportal approach	Unilateral thoracoscopic approach	6
Xu 2020	China	Retrospective cohort	July 2015 to February 2019	37/70	Masaoka stage I–II thymomas, hyperplasia of the thymus gland with myasthenia gravis, and thymic cysts	Subxiphoid and subcostal arch approach	Right-side thoracoscopic approach	6
Yano 2017	Japan	Retrospective cohort	March 2004 to November 2015	14/46	Anterior or mediastinal tumors or myasthenia gravis	Subxiphoid approach	Lateral approach	6
Yoshida 2021	Japan	Retrospective cohort	September 2015 to November 2018	6/5	Anterior mediastinum tumor and myasthenia gravis	Subxiphoid uniportal approach	Median sternotomy	6
Zhang 2019	China	Retrospective cohort	January 2015 to January 2018	28/70	Masaoka stage I–II thymoma without myasthenia gravis	Subxiphoid and subcostal arch approach	Lateral thoracoscopic approach	6

### Primary Outcomes

**Figure 2:**
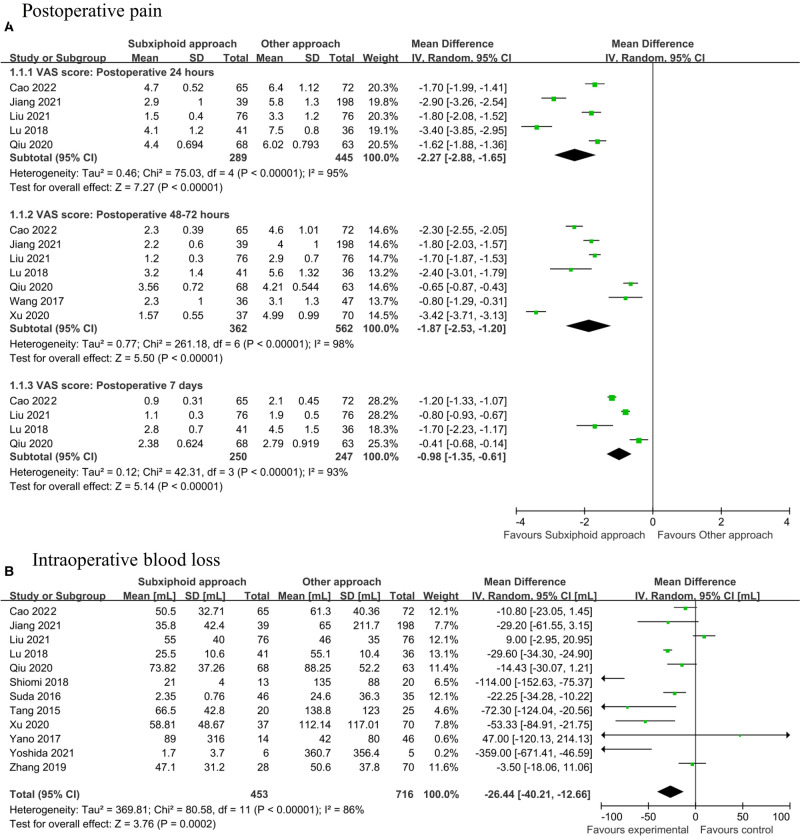
Pooled estimates of included studies evaluating the effect of subxiphoid approach on (**A**) postoperative pain and (**B**) intraoperative blood loss.

**Figure 3:**
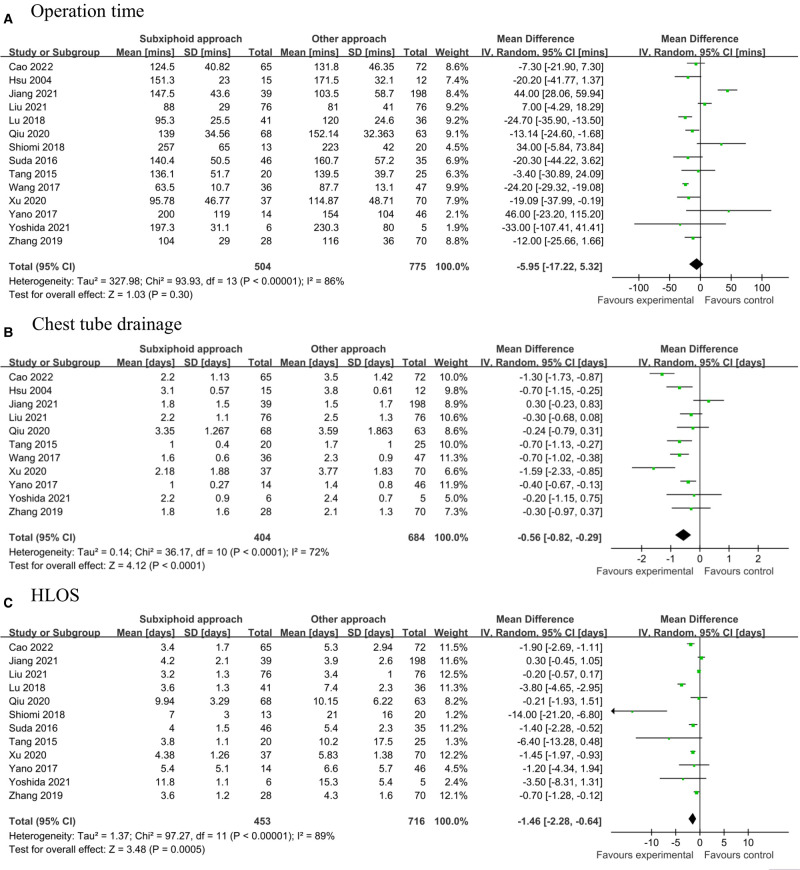
Pooled estimates of included studies evaluating the effect of subxiphoid approach on (**A**) operation time, (**B**) duration of chest tube drainage, and (**C**) HLOS. HLOS, hospital length of stay.

Figure 2A shows the association of the subxiphoid approach with the postoperative pain by comparison with the control group. The pooled estimates revealed that the subxiphoid approach was associated with a significantly decrease in postoperative pain as indicated by visual analog scale (VAS) (WMD: 24 h: −2.27, 95% CI, −2.88 to −1.65, *p* < 0.001; 48–72 h: −1.87, 95% CI, −2.53 to −1.20, *p* < 0.001; 7 days: −0.98, 95% CI, −1.35 to −0.61, *p* < 0.001). Figure 2B showed that patients undergoing subxiphoid approach had less intraoperative blood loss (WMD: −26.44 mL, 95% CI, −40.21 to −12.66, *p* < 0.001). Figure 3 shows the association of the subxiphoid approach with the operation time, duration of chest tube drainage, and HLOS by comparison with the control group. The results showed that no significant difference in operation time (WMD: −5.95 mins, 95% CI, −17.22 to 5.32, *p* = 0.30; Figure 3A) between subxiphoid group and control group were detected, while patients in subxiphoid group had a shorter duration of chest tube drainage (WMD: −0.56 days, 95% CI, −0.82 to −0.29, *p* < 0.001; Figure 3B) and HLOS (WMD: −1.46 days, 95% CI, −2.28 to −0.64, *p* < 0.001; Figure 3C). Funnel plots were used to assess the potential publication bias for primary outcomes of intraoperative blood loss, operation time, duration of chest tube drainage, and HLOS, and possible publication bias was detected by visually inspecting funnel plots (Supplementary Figure S1).

### Subgroup Analysis

Table 2 shows the pooled estimates from sensitivity and subgroup analyses for the primary outcomes. In the sensitivity analysis with the unilateral thoracoscopic approach as the control group, we found that the subxiphoid approach consistently correlated with decreased postoperative pain, shorter duration of chest tube drainage, shorter HLOS, less intraoperative blood loss, but not shortened operation time. The results from the overall analysis hold true in the subgroup analysis of studies performed in China, and the association of the subxiphoid approach with shorter chest tube drainage and HLOS was also observed by meta-analysis of the studies performed in Japan. While the difference in intraoperative blood loss between the subxiphoid group and the control group was not detected, postoperative pain cannot be evaluated in the subgroup analysis of studies performed in Japan.

**Table 2:** Primary outcomes according to subgroup and sensitivity analysis.

Sensitivity analysis	Study	Participant	Mean difference (95%CI)	*I* ^2^	*p* value
Subxiphoid vs. Unilateral thoracoscopic approach					
VAS score: Postoperative 48–72 h	7	924	−1.87 (−2.53 to −1.20)	98%	<0.001
Blood loss (mL)	10	1,125	−18.81 (−30.93 to −6.69)	83%	0.002
Operative time (mins)	12	1,235	−7.34 (−18.82 to 4.15)	88%	0.21
Drainage time (days)	10	1,077	−0.58 (−0.85 to −0.30)	75%	<0.001
HLOS (days)	10	1,125	−1.25 (−2.04 to −0.46)	89%	0.002
Studies performed in China					
VAS score: Postoperative 48–72 h	7	924	−1.87 (−2.53 to −1.20)	98%	<0.001
Blood loss (mL)	8	984	−19.32 (−33.68 to −4.96)	87%	0.008
Operative time (mins)	10	1,094	−7.57 (−19.77 to 4.63)	89%	0.22
Drainage time (days)	9	1,017	−0.60 (−0.93 to −0.28)	76%	<0.001
HLOS (days)	8	984	−1.24 (−2.14 to −0.33)	91%	0.007
Studies performed in Japan					
VAS score: Postoperative 48–72 h	0	0	NA	NA	NA
Blood loss (mL)	4	185	−66.51 (−149.44 to 16.43)	88%	0.12
Operative time (mins)	4	185	5.22 (−32.44 to 42.88)	62%	0.79
Drainage time (days)	2	71	−0.38 (−0.65 to −0.12)	0	0.004
HLOS (days)	4	185	−3.54 (−6.93 to −0.15)	76%	0.04

*NA, not available.*

### Secondary Outcomes

For secondary outcomes, there is no significant difference in the incidence of complications of transition to thoracotomy (RR: 0.94, 95% CI, 0.26–3.41, *p* = 0.13; Supplementary Figure S2A), postoperative pleural effusion (RR: 1.41, 95% CI, 0.65–3.07, *p* = 0.59; Supplementary Figure S2B), phrenic nerve palsy (RR: 0.61, 95% CI, 0.22–1.70, *p* = 0.44; Supplementary Figure S2C), lung infection (RR: 1.08, 95% CI, 0.49–2.41, *p* = 0.48; Supplementary Figure S2D) between subxiphoid group and control group.

## Discussion

### Main Findings

Our meta-analysis by incorporating 14 studies with 1,279 patients indicates that the subxiphoid approach was associated with reduced postoperative pain, shortened chest tube drainage duration, shortened HLOS, and reduced intraoperative blood loss. Besides, it has no impact on operation time and the incidence of complications of transition to thoracotomy, postoperative pleural effusion, phrenic nerve palsy, and lung infection. These findings indicate that the subxiphoid approach is a feasible alternative approach compared with other approaches, even can be a better option for anterior mediastinal surgery.

### Comparison with Previous Studies

The previous two meta-analyses compared the effect of VATS via the subxiphoid approach and lateral VATS in thymectomy, and they consistently suggested that VATS via the subxiphoid approach is a feasible alternative method in thymectomy (23, 24). However, they only explored the effects and safety of the subxiphoid approach for thymectomy in patients with thymoma. Meanwhile, they only compared the subxiphoid thoracoscopic thymectomy to lateral intercostal thoracoscopic thymectomy. While the subxiphoid approach can also be used in some other mediastinal surgeries, and different approaches, but not only the lateral intercostal approach and subxiphoid approach, can be used for mediastinal surgery. Thus, our study expanded the previous findings in the following aspects. First, we focused more on the feasibility of the subxiphoid approach in anterior mediastinal surgery, not only in thymectomy. Patients with anterior mediastinal lesions were all included in our research, and subgroup and sensitivity analyses were performed. Thus, our meta-analysis helps provide evidence to promote the indication of the subxiphoid approach for all anterior mediastinal surgery. Second, subgroup analyses from our study also supported the advantages of the subxiphoid approach compared to the unilateral approach, which means our results were consistent with the previous two meta-analyses. Namely, our study strengthens this previous finding with more studies and an increased sample size of 14 studies and 1,279 patients. Third, more clinical outcomes were investigated in our study. Our meta-analysis collected several meaningful secondary outcomes compared to the previous studies, including the incidence of transition to thoracotomy, postoperative pleural effusion, phrenic nerve palsy, and lung infection. No significant difference was observed for secondary outcomes, indicating that the subxiphoid approach is a good optional approach for anterior mediastinal surgery.

### Potential Mechanism

The benefit of VATS via the subxiphoid approach in anterior mediastinal surgery can be explained by the following aspects. The subxiphoid approach can avoid the compression and injury of the intercostal nerve during operation, and the chest tube via subxiphoid access can further improve this effect (15, 21, 25). Moreover, the subxiphoid approach can also be performed by only one port under the xiphoid, and a reduction in the number of thoracic ports leads to a reduction in damage to the intercostal nerves or muscles (17). The view from the subxiphoid approach is similar to the view from median sternotomy, and a better visual field leads to better identification and dissection of anterior mediastinal vessels such as the brachiocephalic vein and the thymic veins, which can explain the reduction of blood loss (19, 23). Meanwhile, a good visual field can also reduce accidental injury, which shortens the duration of chest tube drainage. Due to the reduction of postoperative pain and the shortening of the duration of chest tube drainage, the patient’s compliance during hospitalization would be strengthened, and patients are more likely to actively cough and expectorate, perform deep breathing exercises, and perform early off-bed ambulation, which helps improve pulmonary function, reduce the incidence of chest infection and promote rapid recovery (23). Naturally, the shortening of HLOS is well explained. There was no significant difference in operation time between the subxiphoid and control groups. This result may be due to the learning curve of VATS via the subxiphoid approach (11). A surgeon skilled in minimally invasive surgical techniques would achieve better surgical outcomes by performing VATS via subxiphoid approach.

### Limitations

Limitations of this meta-analysis should be acknowledged. First, almost all the included studies are retrospective, and the quality of evidence can be affected. Second, although all studies are about VATS via the subxiphoid approach, the methods of implementing the subxiphoid approach are different, and the surgery types of the control group are not the same. These clinical heterogeneities may contribute to high statistical heterogeneity and inconsistent results. However, subgroup and sensitivity analyses were performed, and stable results were observed. Besides, it has been reported that the subxiphoid approach can also be used via awake surgery (26), while none of our included studies investigate this issue, and no further analysis can be performed. Third, the VAS score was incorporated in our meta-analysis to evaluate postoperative pain, but the dosage and total amount of analgesic cannot be investigated. Forth, the medical cost was not reported in all the included studies, which makes the cost-effectiveness of the subxiphoid approach unexplored. Fifth, the prognosis or status of clinical remission of patients with myasthenia gravis was unable to be analyzed because of the limited studies included in the current meta-analysis. Finally, all the included studies were accomplished in China or Japan, and it is uncertain whether the conclusion of this meta-analysis can be generalized to other regions of the world.

## Conclusions

VATS via subxiphoid approach is a feasible alternative approach compared with other approaches, even can be a better option for anterior mediastinal surgery. Considering the heterogeneity and limitations, these findings need to be interpreted with caution, and further large-scale multicenter randomized controlled trials are needed.

## Data Availability

The original contributions presented in the study are included in the article/Supplementary Material, further inquiries can be directed to the corresponding author/s.
