# Supplementary material for: Feasibility of Video-Assisted Thoracoscopic Surgery via Subxiphoid Approach in Anterior Mediastinal Surgery: A Meta-Analysis
Source: Front Surg. 2022 May 6;9:900414. doi: 10.3389/fsurg.2022.900414 (PMC9122262; doi:10.3389/fsurg.2022.900414)
Supplement: Supplementary file 1 [file fsurg-09-900414_Table_1_v2.docx]

| **Table S1** Description of the subxiphoid and control approaches | | | |
| --- | --- | --- | --- |
| **Study ID** | **Subxiphoid approach** | **Number of ports** | **Control** |
| Cao 2022 | A 3-cm incision at 1.5 cm below the lower edge of the xiphoid and two 5-mm ports under the bilateral costal arch. | **3** | A 3-cm observation port in the 7th intercostal space at the mid-axillary line and an operation port in the 5th intercostal space at anterior line. |
| Hsu 2004 | A 6-cm semi-curved incision below the xiphoid and two 1-cm ports at the bilateral anterior axillary line in the ﬁfth or sixth intercostal space. | **3** | Initial port site at the right posterior axillary line in the sixth intercostal space for insertion of the thoracoscope. Additional port sites along the anterior axillary lines in proper intercostal spaces. |
| Jiang 2021 | A 3-cm incision below the xiphoid and two 5-mm ports at the midclavicular line intersecting with the bilateral costal arch. | **3** | Three 10-mm ports were created. |
| Liu 2021 | A 3-cm transverse incision at about 1 cm below the lower edge of the xiphoid. | **1** | A 3-cm incision ﬁfth intercostal space along the anterior axillary line. |
| Lu 2018 | A 3-cm incision below the xiphoid and two 5-mm ports at the midclavicular line intersecting with the bilateral costal arch. | **3** | Initial port site at the right posterior axillary line in the sixth intercostal space for insertion of the thoracoscope. Additional port sites along the anterior axillary lines in proper intercostal spaces. |
| Qiu 2020 | A 2-cm straight incision in the middle of the xiphoid and two 5-mm ports under the bilateral costal arch. | **3** | One observation port in the 4th intercostal space at the right axillary midline, and two operation ports in the 3rd and 5th intercostal spaces hole at the anterior axillary line. |
| Shiomi 2018 | A 2.5-cm longitudinal incision below the xiphoid and a 5-mm port at 5th intercostal space on the lateral side of the midclavicular line. | **2** | Extended thymectomy via sternotomy. |
| Suda 2016 | A 3-cm transverse incision at about 1 cm below the lower edge of the xiphoid | **1** | Three 1-cm skin incisions with 1 on anterior axillary line in the third intercostal space and 2 on the midaxillary line in the third and sixth intercostal spaces. |
| Tang 2015 | A 2.5-cm incision at the lower edge of the xiphoid and two 5-mm ports at the intersection point of bilateral midclavicular line and subcostal margin. | **3** | Three 5 to 10 mm incisions on the right chest. |
| Wang 2017 | Three subxiphoid incisions (A 12-mm incision and two 5-mm ports). | **3** | Unilateral 3-port thoracoscopic approach. |
| Xu 2020 | A 3-cm incision below the xiphoid and two 5-mm ports at the midclavicular line intersecting with the bilateral costal arch. | **3** | One incision at the fifth intercostal space on the right posterior axillary line, two additional 5-mm ports at the third intercostal space on the anterior axillary line and at the fifth intercostal space on middle clavicular line. |
| Yano 2017 | A 2.5–3-cm transverse skin incision at 1–2 cm below the xiphoid and three 10-mm ports. | **4** | Six ports and a cervical incision were necessary to complete total thymectomy in the lateral approach. |
| Yoshida 2021 | A 3-cm transverse incision at about 1 cm below the lower edge of the xiphoid. | **1** | Extended thymectomy via sternotomy. |
| Zhang 2019 | A 3-cm incision at 1.5 cm below the lower edge of the xiphoid and two 5-mm ports under the bilateral costal arch. | **3** | One 0.5–1.0 cm observation port in the 6th intercostal space at the anterior axillary line and two main operation ports in the 3rd intercostal space at the anterior axillary and in the 5th intercostal space at the midclavicular line. |
