# Supplementary material for: Feasibility of Video-Assisted Thoracoscopic Surgery via Subxiphoid Approach in Anterior Mediastinal Surgery: A Meta-Analysis
Source: Front Surg. 2022 May 6;9:900414. doi: 10.3389/fsurg.2022.900414 (PMC9122262; doi:10.3389/fsurg.2022.900414)
Supplement: Supplementary file 2 [file fsurg-09-900414_Table_2_v2.docx]

| **Table S2** Outcome data of primary outcomes of postoperative pain and blood loss according subxiphoid and other approaches. | | | | | | | | | |
| --- | --- | --- | --- | --- | --- | --- | --- | --- | --- |
| **Study ID** | **No.** | **Postoperative 24 h VAS** | | **Postoperative 72 h VAS** | | **Postoperative 7day VAS** | | **Blood loss (mL)** | |
|  | **Subxiphoid vs. Control** | **Subxiphoid** | **Control** | **Subxiphoid** | **Control** | **Subxiphoid** | **Control** | **Subxiphoid** | **Control** |
| Cao 2022 | 65/72 | 4.7 ± 0.52 | 6.4 ± 1.12 | 2.3 ± 0.39 | 4.6 ± 1.01 | 0.9 ± 0.31 | 2.1 ± 0.45 | 50.5 ± 32.71 | 61.3 ± 40.36 |
| Hsu 2004 | 15/12 | NA | NA | NA | NA | NA | NA | NA | NA |
| Jiang 2021 | 39/198 | 2.9 ± 1 | 5.8 ± 1.3 | 2.2 ± 0.6 | 4 ± 1 | NA | NA | 35.8 ± 42.4 | 65 ± 211.7 |
| Liu 2021 | 76/76 | 1.5 ± 0.4 | 3.3 ± 1.2 | 1.2 ± 0.3 | 2.9 ± 0.7 | 1.1 ± 0.3 | 1.9 ± 0.5 | 55 ± 40 | 46 ± 35 |
| Lu 2018 | 41/36 | 4.1 ± 1.2 | 7.5 ± 0.8 | 3.2 ± 1.4 | 5.6 ± 1.3 | 2.8 ± 0.7 | 4.5 ± 1.5 | 25.5 ± 10.6 | 55.1 ± 10.4 |
| Qiu 2020 | 68/63 | 4.4±0.694 | 6.02±0.793 | 3.56±0.72 | 4.21±0.544 | 2.38±0.624 | 2.79±0.919 | 73.82±37.26 | 88.25±52.20 |
| Shiomi 2018 | 13/20 | NA | NA | NA | NA | NA | NA | 21 ± 4 | 135 ± 88 |
| Suda 2016 | 46/35 | NA | NA | NA | NA | NA | NA | 2 (2 to 3) **^#^** | 20 (3.0 to 50.0) **^#^** |
| Tang 2015 | 20/25 | NA | NA | NA | NA | NA | NA | 66.5±42.8 | 138.8±123.0 |
| Wang 2017 | 36/47 | NA | NA | 2.3 ± 1.0 | 3.1 ± 1.30 | NA | NA | NA | NA |
| Xu 2020 | 37/70 | NA | NA | 1.57 ± 0.55 | 4.99 ± 0.99 | NA | NA | 58.81 ± 48.67 | 112.14 ± 117.01 |
| Yano 2017 | 14/46 | NA | NA | NA | NA | NA | NA | 89 ± 316 | 42 ± 80 |
| Yoshida 2021 | 6/5 | NA | NA | NA | NA | NA | NA | 1.7 ± 3.7 | 360.7 ± 356.4 |
| Zhang 2019 | 28/70 | NA | NA | NA | NA | NA | NA | 40 (30–70)**^#^** | 40 (30–80)**^#^** |
| VAS, Visual Analogue Scale.  ^#^ median (IQR) | | | | | | | | | |
