# Supplementary material for: Feasibility of Video-Assisted Thoracoscopic Surgery via Subxiphoid Approach in Anterior Mediastinal Surgery: A Meta-Analysis
Source: Front Surg. 2022 May 6;9:900414. doi: 10.3389/fsurg.2022.900414 (PMC9122262; doi:10.3389/fsurg.2022.900414)
Supplement: Supplementary file 3 [file fsurg-09-900414_Table_3_v2.docx]

| **Table S3** Outcome data of primary outcomes of operation time, duration of chest drainage, and HLOS according subxiphoid and other approaches. | | | | | | | |
| --- | --- | --- | --- | --- | --- | --- | --- |
| **Study ID** | **No.** | **Operation time (mins)** | | **Duration of chest drainage (days)** | | **HLOS (days)** | |
|  | **Subxiphoid *vs.* Control** | **Subxiphoid** | **Control** | **Subxiphoid** | **Control** | **Subxiphoid** | **Control** |
| Cao 2022 | 65/72 | 124.5 ± 40.82 | 131.8 ± 46.35 | 2.2 ± 1.13 | 3.5 ± 1.42 | 3.4 ± 1.70 | 5.3 ± 2.94 |
| Hsu 2004 | 15/12 | 151.3 (120 - 200)* | 171.5 (120 - 225)* | 3.1 (2 - 4 )* | 3.8 (2 - 4)* | NA | NA |
| Jiang 2021 | 39/198 | 147.5 ± 43.6 | 103.5 ± 58.7 | 1.8 ± 1.5 | 1.5 ± 1.7 | 4.2 ± 2.1 | 3.9 ± 2.6 |
| Liu 2021 | 76/76 | 88 ± 29 | 81 ± 41 | 2.2 ± 1.1 | 2.5 ± 1.3 | 3.2 ± 1.3 | 3.4 ± 1.0 |
| Lu 2018 | 41/36 | 95.3 ± 25.5 | 120.0 ± 24.6 | 0** | 3.6 ± 1.2 | 3.6 ± 1.3 | 7.4 ± 2.3 |
| Qiu 2020 | 68/63 | 139±34.56 | 152.14±32.363 | 3.35±1.267 | 3.59±1.863 | 9.94±3.29 | 10.15±6.22 |
| Shiomi 2018 | 13/20 | 257 ± 65 | 223 ± 42 | NA | NA | 7 ± 3 | 21 ± 16 |
| Suda 2016 | 46/35 | 139 (108-174) **^#^** | 150 (128 to 202) **^#^** | NA | NA | 4 (3-5) **^#^** | 5 (4-7) **^#^** |
| Tang 2015 | 20/25 | 136.1±51.7 | 139.5±39.7 | 1.0±0.4 | 1.7±1.0 | 3.8±1.1 | 10.2±17.5 |
| Wang 2017 | 36/47 | 63.5 ± 10.7 | 87.7 ± 13.1 | 1.6 ± 0.6 | 2.3 ± 0.9 | NA | NA |
| Xu 2020 | 37/70 | 95.78 ± 46.77 | 114.87 ± 48.71 | 2.18 ± 1.88 | 3.77 ± 1.83 | 4.38 ± 1.26 | 5.83 ± 1.38 |
| Yano 2017 | 14/46 | 200 ± 119 | 154 ± 104 | 1.0 ± 0 | 1.4 ± 0.8 | 5.4 ± 5.1 | 6.6 ± 5.7 |
| Yoshida 2021 | 6/5 | 197.3 ± 31.1 | 230.3 ± 80.0 | 2.2 ± 0.9 | 2.4 ± 0.7 | 11.8 ± 1.1 | 15.3 ± 5.4 |
| Zhang 2019 | 28/70 | 104 ± 29 | 116 ± 36 | 1.8 ± 1.6 | 2.1 ± 1.3 | 3.6 ± 1.2 | 4.3 ± 1.6 |
| **^#^** median (IQR) * Mean (Range) ** No chest tube | | | | | | | |
