# Supplementary material for: Feasibility of Video-Assisted Thoracoscopic Surgery via Subxiphoid Approach in Anterior Mediastinal Surgery: A Meta-Analysis
Source: Front Surg. 2022 May 6;9:900414. doi: 10.3389/fsurg.2022.900414 (PMC9122262; doi:10.3389/fsurg.2022.900414)
Supplement: Supplementary file 4 [file fsurg-09-900414_Table_4_v2.docx]

| **Table S4** Outcome data of secondary outcomes according subxiphoid and Control approaches | | | | | | | | | |
| --- | --- | --- | --- | --- | --- | --- | --- | --- | --- |
| **Study ID** | **No.** | **Conversion** | | **Pleural effusion** | | **Phrenic nerve palsy** | | **Pulmonary infection** | |
|  | **Subxiphoid *vs.* Control** | **Subxiphoid** | **Control** | **Subxiphoid** | **Control** | **Subxiphoid** | **Control** | **Subxiphoid** | **Control** |
| Cao 2022 | 65/72 | 1/65 | 2/72 | 1/65 | 2/72 | 0/65 | 1/72 | 2/65 | 3/72 |
| Hsu 2004 | 15/12 | NA | NA | NA | NA | NA | NA | NA | NA |
| Jiang 2021 | 39/198 | 3/39 | 2/198 | 5/39 | 13/198 | NA | NA | 3/39 | 4/198 |
| Liu 2021 | 76/76 | NA | NA | 2/76 | 3/76 | 0/76 | 3/76 | 0/76 | 1/76 |
| Lu 2018 | 41/36 | 2/41 | 4/36 | 1/41 | 0/36 | 0/41 | 1/36 | NA | NA |
| Qiu 2020 | 68/63 | 1/68 | 2/63 | NA | NA | 1/68 | 0/63 | 3/68 | 4/63 |
| Shiomi 2018 | 13/20 | NA | NA | NA | NA | NA | NA | NA | NA |
| Suda 2016 | 46/35 | NA | NA | NA | NA | 1/46 | 2/35 | NA | NA |
| Tang 2015 | 20/25 | NA | NA | NA | NA | NA | NA | NA | NA |
| Wang 2017 | 36/47 | 0/36 | 1/47 | NA | NA | NA | NA | NA | NA |
| Xu 2020 | 37/70 | NA | NA | NA | NA | 3/37 | 0/70 | 0/36 | 2/70 |
| Yano 2017 | 14/46 | NA | NA | NA | NA | 0/14 | 6/46 | NA | NA |
| Yoshida 2021 | 6/5 | NA | NA | NA | NA | NA | NA | NA | NA |
| Zhang 2019 | 28/70 | NA | NA | NA | NA | 0/28 | 2/70 | 0/28 | 1/70 |
